# High rates of observed face mask use at Colorado universities align with students’ opinions about masking and support the safety and viability of in-person higher education during the COVID-19 pandemic

**DOI:** 10.1186/s12889-023-15211-y

**Published:** 2023-02-09

**Authors:** Kevin C. Clark, Maximilian J. Bailey, Stefan Wasshuber, Raissa Huntley, Kristen K. Bjorkman, Leisha Conners Bauer, Camille L. Paige, Sara L. Sawyer, Michaila Czarnik, Margaret A. Riggs, Margaret J. Gutilla, Tanya L. Alderete

**Affiliations:** 1 Department of Integrative Physiology, University of Colorado, Boulder, CO United States of America; 2 Colorado School of Public Health, Colorado State University, Fort Collins, CO United States of America; 3 BioFrontiers Institute, University of Colorado, Boulder, CO United States of America; 4 Health and Wellness Services, University of Colorado, Boulder, CO United States of America; 5 CDC COVID-19 Emergency Response Team, Atlanta, GA United States of America

**Keywords:** United States, Colleges, Universities, Institutes of higher education, Mask Use, COVID-19, Student opinions, Student Health, Mask adherence, Mask mandates, MASCUP!, SARS-CoV-2, Prevention

## Abstract

**Background:**

Over the course of the COVID-19 pandemic, colleges and universities have focused on creating policies, such as mask mandates, to minimize COVID-19 transmission both on their campuses and in the surrounding community. Adherence to and opinions about these policies remain largely unknown.

**Methods:**

The Centers for Disease Control and Prevention (CDC) developed a cross-sectional study, the Mask Adherence and Surveillance at Colleges and Universities Project (MASCUP!), to objectively and inconspicuously measure rates of mask use at institutes of higher education via direct observation. From February 15 through April 11, 2021 the University of Colorado Boulder (CU, n = 2,808 observations) and Colorado State University Fort Collins (CSU, n = 3,225 observations) participated in MASCUP! along with 52 other institutes of higher education (n = 100,353 observations) spanning 21 states and the District of Columbia. Mask use was mandatory at both Colorado universities and student surveys were administered to assess student beliefs and attitudes.

**Results:**

We found that 91.7%, 93.4%, and 90.8% of persons observed at indoor locations on campus wore a mask correctly at University of Colorado, Colorado State University, and across the 52 other schools, respectively. Student responses to questions about masking were in line with these observed rates of mask use where 92.9% of respondents at CU and 89.8% at CSU believe that wearing masks can protect the health of others. Both Colorado universities saw their largest surges in COVID-19 cases in the fall of 2020, with markedly lower case counts during the mask observation window in the spring of 2021.

**Conclusion:**

High levels of mask use at Colorado’s two largest campuses aligned with rates observed at other institutes across the country. These high rates of use, coupled with positive student attitudes about mask use, demonstrate that masks were widely accepted and may have contributed to reduced COVID-19 case counts. This study supports an emerging body of literature substantiating masks as an effective, low-cost measure to reduce disease transmission and establishes masking (with proper education and promotion) as a viable tactic to reduce respiratory disease transmission on college campuses.

**Supplementary Information:**

The online version contains supplementary material available at 10.1186/s12889-023-15211-y.

## Background

Severe acute respiratory syndrome coronavirus 2 (SARS-CoV-2), the virus that causes Coronavirus Disease of 2019 (COVID-19), is transmitted by respiratory droplets that are exhaled by those carrying virus when they cough, sneeze, talk, or breathe [1–4]. Masks are primarily intended to reduce transmission by catching infectious respiratory droplets from a person who is ill (source control) and, perhaps more importantly, asymptomatic and presymptomatic persons as well. These “silent” transmissions (from presymptomatic and asymptomatic individuals) have been estimated to be responsible for more than 50% of the new infections in COVID-19 outbreaks [5] and silent transmission alone can sustain outbreaks even if all symptomatic cases are immediately isolated [5]. Additionally, viral load has been shown to be associated with likelihood of transmission [6, 7], yet there are no differences in the distribution of viral load between positive asymptomatic and positive symptomatic populations [8–13] supporting that asymptomatic individuals can transmit SARS-CoV-2 as readily as those who are sick with COVID-19. Well-fitting face masks can reduce inhalation of these droplets by the wearer (wearer protection) in addition to providing source control. The community benefit of masking for the control of SARS-CoV-2 transmission comes from the combination of wearer protection and source control, regardless of symptomatic status, and this benefit increases with growing numbers of people using masks consistently and correctly [14, 15].

A recent study with a sample size of approximately 20 million has empirically evaluated that mask wearing alone has the potential to reduce effective reproduction number *R* of SARS-CoV-2 transmission by as much as 25% [16]. Additional epidemiological investigations have helped quantify the benefit of mask wearing to prevent the spread of SARS-CoV-2, with multiple studies finding a reduced risk of infection for persons wearing masks (70–100% reduction) when exposed to people confirmed to be carrying SARS-CoV-2 [17–20] and a reduced disease incidence in counties with a mask mandate versus without [21]. Another study looked at 15 states and Washington DC, which all mandated public mask use, and found that mask use in public was associated with a significant decline in the daily COVID-19 growth rate by 0.9% 1–5 days after the mandate was signed. This grew to a 2.0% reduction in the daily growth rate after 21 or more days following the mandate [22]. In consideration of the evidence in favor of mask use many college campuses and institutes of higher education across the United States instituted mask mandates in public areas on campus and when outdoors within six feet of others during the 2020–2021 academic year. Supporting this, studies have found higher rates of mask wearing on college campuses within counties or states with mask mandates [23–26].

During the 2020–2021 academic year, the decision to return to in-person learning was controversial [27, 28] and remains an important topic and consideration for education amidst future COVID-19 outbreaks and other possible pandemics. While some coursework may have been adapted for remote instruction with minimal loss in quality, other types of education suffered substantially in a remote format [29]. Furthermore the COVID-19 pandemic has exacerbated pre-existing educational inequalities; the transition to remote learning has disproportionately impacted low-income households, people with disabilities, and females who were all less likely to access remote learning than their peers [30, 31]. A return to in-person education and some elements of “normalcy” were essential for the educational experience, social development, and emotional well-being of college students [32–34]. For these reasons, and in consideration of financial pressures, colleges and universities enacted mitigation practices to allow campuses to reopen and resume classroom instruction. For example, during the 2020–2021 academic year, the University of Colorado Boulder (CU) and Colorado State University Fort Collins (CSU) had policies in place that mandated mask usage indoors and recommended mask usage outdoors when maintaining over six feet of physical distance was not possible. These recommendations were in line with the Centers for Disease Control and Prevention’s (CDC) recommendations for masking at institutes of higher education. Additionally, CU and CSU developed aggressive surveillance/monitor testing measures to identify and proactively isolate individuals with COVID-19 and trace their related contacts. Both Colorado universities also mandated that masks be worn everywhere in dorms (except in private rooms) and that dining halls provide grab-and-go options for students to avoid eating inside *en masse*. These measures, in combination with strategic holidays and academic schedules, were put in place to mitigate and minimize the impact of in-person education on the rates of COVID-19 transmission on campus and in the neighboring community.

As of 2018, approximately 41% of adults aged 18–24 years were enrolled in a college or university [35] and a projected 22 million people are expected to be enrolled in college in the year 2022 [36]. The return of students and faculty to college campuses in Fall of 2020 was coupled with some scrutiny as some felt this move posed a potential public health risk which highlighted the need to examine adherence to masking recommendations at institutes of higher education. From a public health standpoint, it is critical to understand masking behaviors and attitudes on college campuses to effectively prevent the spread of SARS-CoV-2 and tailor public health messaging. A self-reported study found that adults aged 18–29 years used a mask 69.6–86.1% of the time [37]. However, there is still a need to objectively quantify mask usage and behavior on college campuses. Direct observation has been established as the standard way to assess infection prevention and control recommendations, such as handwashing compliance in healthcare settings [38]. The CDC utilized this methodology and translated it to developing a protocol for the Mask Adherence Surveillance at Colleges and Universities Project (MASCUP!) study. The observations and masking data collected for the current study are part of MASCUP!, which objectively recorded mask adherence on college campuses and in nearby areas off campus. The CDC conducted the first observational study to document mask wearing in the fall of 2020, and found that an average of 94.8% of people wore masks indoors on campus across six universities [24]. CU and CSU represent two campuses of 54 nationally that participated in MASCUP! in the spring of 2021. The primary aim of our study was to investigate mask usage at two universities in Colorado (CU and CSU) and compare observations at these two campuses with other participating universities across the nation (locations pictured in **S1 Fig**). As a secondary aim we sought to describe our observations in the context of surveyed student opinions, COVID-19 case data, vaccination rates and the public health policies that were in place. This quantitative assessment of masking behavior at the two largest universities in Colorado (n = 6,033 observations) along with 52 other universities across the nation (n = 100,353 observations) provides a data-driven perspective of mask use at institutes of higher education and has the potential to inform public health and education policies. This investigation is the first of its kind to pair objective data on mask use from college campuses with reports of student opinions and perspective about masking during the same time period.

## Methods

### Mask observation

Observations took place on the campuses of CU and CSU, and in the immediately surrounding community, from February 15, 2021 through April 11, 2021. Student observers (n = 13 at CU; n = 8 at CSU) were trained on data collection procedures using standardized CDC protocols [39] that were determined by each Colorado university’s respective Institutional Review Boards (IRB) to be exempt from IRB approval. The protocol and sampling methodology provided by CDC were based on *Resolve to Save Lives*, an initiative promoting the measuring and adopting of face mask use to reduce the transmission of COVID-19 [40]. The training materials and the protocol and sampling methodology were standardized and consistent across all institutes participating in MASCUP!. The standardized methods were intentional to allow for comparison between universities and with the aggregate of other schools in the study. Observers remained inconspicuous and did not interact with persons they were observing. During an observation session the observer also wore a mask correctly per CDC guidelines (cloth, surgical, or N95 mask over the nose and secured under the chin).

At CU, observations were split between indoor and outdoor locations over the course of eight weeks (1,509 of 2,808 observations were outdoors). Observers tracked mask usage on varying days (Monday – Saturday) and times (8am–7pm) from fixed sites on the campus (e.g., student centers, residence halls, recreation centers (gyms), libraries, academic buildings, dining facilities, and outdoor common areas) and at nearby public locations on “The Hill” (the main off-campus student neighborhood near campus). Data collection at both universities, and in the larger aggregated study sample, occurred in places with no expectation of privacy. An “observation session” was defined as a period during which observations took place at one defined location. At CU, on average 351 observations were collected per week by the 13 observers.

At CSU, most observations were performed indoors, with 128 of 3,225 observations being collected outdoors, and observations were only done on campus. However, CSU’s outdoor observations were collected at a high-traffic intersection near the edge of campus with a high volume of foot traffic passing between campus and the adjacent, off-campus area. Therefore, while this location at CSU is technically at the edge of campus, for the purposes of this analysis it was off campus and compared to the off-campus locations observed at CU. On average, CSU collected 403 observations per week by the 8 observers.

For an observation session, each observer was instructed to record every third person that passed by an observation point up to 40 observations at a single location or to perform observations for one hour in duration, whichever came first. Correct mask use was recorded if the mask completely covered the nose and mouth and was also secured under the chin. Observers recorded: (1) whether a mask was worn, and if worn, (2) whether the mask was worn correctly, and (3) the type of mask (i.e., cloth, surgical which includes KN95 type, gaiter, N95 type, or other). If an observer could not visualize the entire mask, then the mask use was recorded as “unknown”. For our analysis, “unknown” observations were excluded and comprised 0.21%, 0.52%, and 0.29% of CU, CSU, and the national aggregate’s data, respectively (CU n = 6, CSU n = 17, National Aggregate n = 287). If traffic was high during a given observation session, the observers were instructed to select every tenth person for the observation period. The every tenth person sampling strategy was not utilized in CU or CSU’s observations but was utilized for some observations at other universities included in the aggregate.

Observers sought to capture the prevailing behaviors from each social group (e.g., group of friends) that was sampled. If the third person observed passing by fell on a cluster of persons, then one observation was recorded for every three people in the group. For example, if the observed group had six individuals and three of which were properly wearing masks, then two observations would be recorded, one for wearing a mask and one for not wearing a mask. Observations were not limited to students; however, in the areas where observations were performed it is reasonable to assume that students were the majority of people observed. On both CU and CSU’s campuses non-students rarely travel through the campus. Students, faculty, and staff are the general population present at the locations where observations were performed and students largely outnumber staff and faculty, even under more limited in-person campus operations.

#### Data collection

Data collection was standardized through the common CDC training materials mentioned and a data collection form was provided to all observers to record in the field. This uniform structure allowed for comparison between institutions involved in this national study. After an observation session, the observer took their observation form and observation data were entered into a survey, collected, and managed using REDCap (Research Electronic Data Capture) tools hosted at Vanderbilt University (Version 9.7) [41, 42]. Over the course of the study, observation data from the respective schools were assembled and returned on a weekly basis by CDC. Data included the counts of persons observed wearing masks, wearing masks correctly, the most common type of mask worn, common errors observed, the location, and the observation point (entry/exit/lobby (indoors), hallway/rooms (indoors), or outdoors) of each session. Every week, study staff members at each university performed quality control of this data and communicated appropriate corrections to CDC. All data corrections that were made were confirmed with mutual agreement between two independent analysts, one from CDC and one from either CU or CSU for their respective data corrections.

### Campus and community context

To characterize the context of our mask observations, we also examined campus contextual factors including COVID-19 testing, positive cases, vaccination rates, and student opinions measured via student surveys. During the study, 53 of the 54 institutes of higher education, including both Colorado campuses, required mask use on campus.

#### Student surveys

**Fig. 1:**
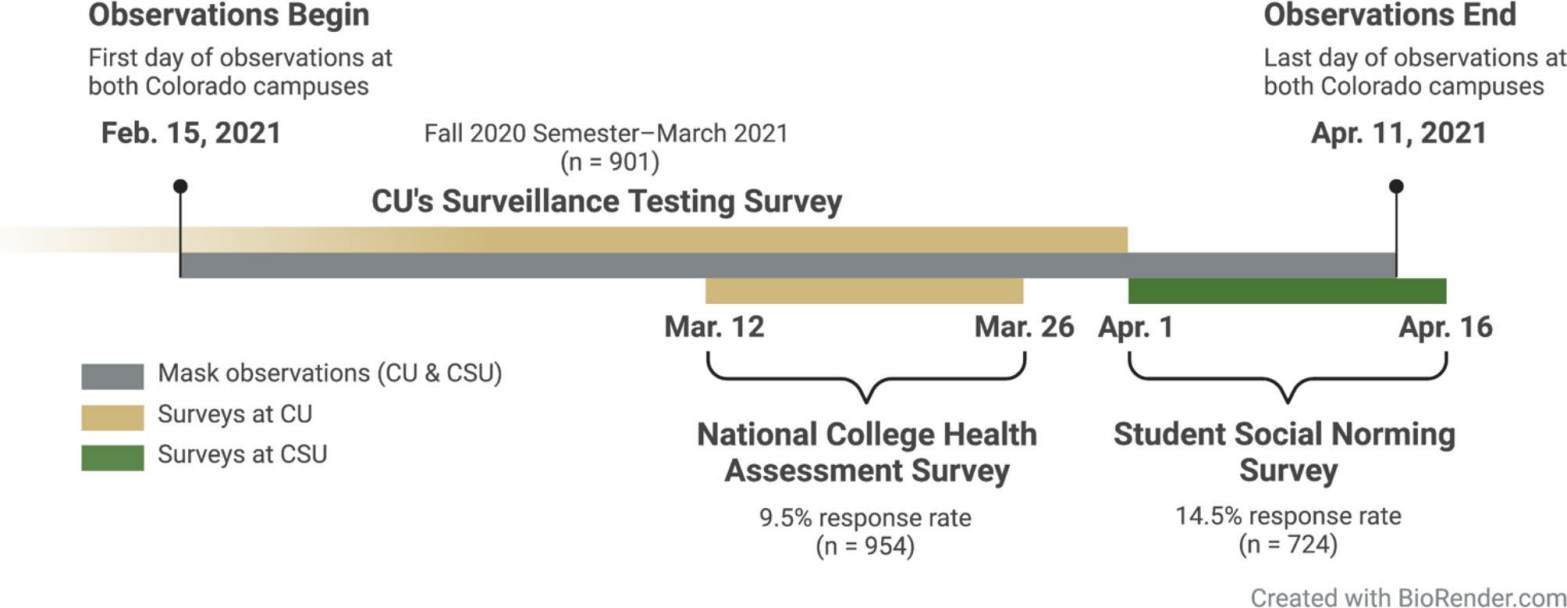
**Timeline of mask observation and student surveys at University of Colorado Boulder (CU) and Colorado State University Fort Collins (CSU).** The National College Health Assessment survey at CU and the Social Norming Survey at CSU were temporally close during the Spring 2021 Semester and are used herein to compare opinions about mask use on each campus. The Surveillance Testing Survey at CU is unique in that it paired individual’s responses with their COVID-19 surveillance test results, giving insight into how different reported behaviors may associate with having tested positive for COVID-19 at any point during the 2020–2021 academic year.

CU and CSU administered multiple surveys over the 2020–2021 academic year assessing student perceptions and feelings about COVID-19. Of particular interest, some survey questions asked about masking and opinions and adherence to public health policies for infection prevention. Here we will outline some surveys with results that complement and offer context for our masking behaviors study. Health Promotion, part of Health and Wellness Services, at CU administered the National College Health Assessment III (NCHA III) survey by the American College Health Association (ACHA) [43]. This survey was distributed to a random sample of 10,000 students (including graduate students) from March 12 to 26, 2021, and had 954 responses for a 9.5% response rate. CU also conducted an additional Surveillance Testing Survey over the course of the fall and spring semesters where CU students and affiliates that tested through the campus saliva-based surveillance testing program could elect to complete an IRB-approved questionnaire (n = 901 people enrolled). This questionnaire data was paired with the saliva-based SARS-CoV-2 RT-qPCR test results to determine if a participant tested positive at any point during the campus surveillance testing program from August 2020 to March 2021. At CSU, the Student Social Norming Task Force administered a Spring Semester Social Norming Survey from April 1 to 16, 2021 to a random sample of 5,000 undergraduate and graduate students with a total of 724 students responding (14.5% response rate). This survey was a follow-up to a similar social norming survey that CSU administered in both the summer and fall of 2020. Vaccination rates reported were determined from self-reported responses in the NCHA III survey (CU) and the Social Norming Survey (CSU) (Fig. 1).

#### COVID-19 testing and case identification

During the 2020–2021 academic year, CU and CSU deployed robust saliva-based surveillance/monitoring testing for SARS-CoV-2 [13] and used FDA approved PCR diagnostic tests to confirm positive surveillance cases. Students, staff, and faculty were recommended for diagnostic testing if they had a positive saliva monitoring test result, had a confirmed exposure via self-report or contact tracing, and/or were evaluated by the care team as potentially having COVID-19. CU started monitoring broadly with the saliva test on the first day of the fall semester and collaborated with CSU to share their collection operation and laboratory methods. Later in the fall, CSU implemented their monitoring system. Both universities had wastewater surveillance from the beginning of Fall 2020. CU and CSU’s diagnostic testing data were downloaded from the publicly available reporting dashboards of the respective universities. All testing data reported are publicly available and herein we report the results of 27,232 COVID-19 diagnostic tests at CU [44, 45] and 21,545 tests at CSU [46] from August 31, 2020 to May 7, 2021.

### Statistical analysis

Descriptive statistics were performed using data from our eight-week study. Frequencies, percentages, and ranges were calculated for mask use (mask vs. no mask), type of mask worn (cloth, surgical, N95, etc.), and correct mask use (correct vs. incorrect vs. no mask) by location and observation point. Correct masking proportions were calculated as the percentage of people wearing masks correctly divided by the total number of people observed. “Unknown” observations were excluded from the analysis and from the denominators of all proportions. Chi-squared tests were used to compare mask use in a variety of different settings, like indoors vs. outdoors and on-campus vs. off-campus. Where comparisons were between two explanatory and two response categories (i.e., indoors vs. outdoors by mask vs. no mask) a Yates’ continuity correction was applied. Chi-squared tests were also used to compare mask use and correct mask use at CU, CSU, and across the 52 other universities (without CU and CSU) collecting data over the eight-week period from February 15 to April 11 of 2021. Lastly, a logistic regression model was constructed to determine if there were differences in masking between CU and CSU while adjusting for observation location (on or off campus, indoors or outdoors) and observation week. P-values and adjusted p-values of < 0.05 were considered statistically significant. A Bonferroni adjustment for multiple testing was made for Chi-squared post-hoc tests. All analyses were conducted using the statistical programming language R version 4.1.2 [47] and RStudio [48]. Lastly, all study activities, analysis, and results were reviewed by CDC and were conducted consistent with applicable local and federal laws and CDC policies (45 C.F.R. part 46; 21 C.F.R. part 56; 42 U.S.C. Section 241(d); 5 U.S.C. Sect. 552a; 44 U.S.C. Section 3501 et seq.).

## Results

During the eight weeks of data collection, a total of 2,808 persons were observed at CU, 3,225 at CSU, and 100,353 at the 52 other universities that participated in the study nationally (Table 1). When comparing observations between the universities and nationally, CU had a larger proportion of observations outdoors and off campus than CSU and the national aggregate. At CU, 46.3% (n = 1,299) of observations took place indoors on campus, 24.9% (n = 701) took place outdoors on campus, and 28.8% (n = 808) were outdoors off campus (Table 2). Whereas at CSU, 96.0% (n = 3,097) of observations took place indoors on campus, and 4.0% (n = 128) took place outdoors off campus. Across all other participating universities, 62.7% (n = 62,971) of observations took place indoors on campus, 19.5% (n = 19,557) were outdoors on campus, and 8.69% (n = 8,721) occurred outdoors off campus.

**Table 1:** **Observed counts and percentages of persons wearing face masks and wearing them correctly at Colorado Universities, by selected characteristics; Feb. 15–Apr. 11, 2021.**

Characteristic	CU Boulder		CSU		National Aggregate	
**Overall mask use**	2,808*		3,225*		100,353*	
Mask Worn	2,492	88.7%	3,138	97.3%	94,280	93.9%
Mask Worn Correctly	2,229	79.4%	2,970	92.1%	86,238	85.9%
**Type of mask**						
Cloth	1,577	63.3%	2,165	69.0%	57,975	61.5%
Surgical§	776	31.1%	853	27.2%	29,297	31.1%
Gaiter	65	2.6%	71	2.3%	3,989	4.2%
N95 type	70	2.8%	45	1.4%	2,732	2.9%
Other	4	0.2%	4	0.1%	285	0.3%
**Location**						
Indoors	1,299	46.3%	3,097	96.0%	72,075	71.8%
Outdoors	1,509	53.7%	128	4.0%	28,278	28.2%
**Campus**						
On campus	2,000	71.2%	3,097	96.0%	82,528	82.2%
Nearby, off campus	808	28.8%	128	4.0%	17,825	17.8%

* All “unknown” observations were excluded. “Unknown” was reported if it could not be determined if the person was wearing a mask or not i.e., if the observer could only see the back of the person’s head.

§ KN95 type masks would be classified as surgical in our training materials, with N95 type capturing respirators with straps that go around the head.

**Table 2:** **Observed overall counts and percentages of persons wearing face masks indoors and outdoors and wearing face masks indoors and outdoors correctly between various campus environments; Feb. 15–Apr. 11, 2021.**

CU Boulder	Total Observed		On Campus		Nearby, Off Campus	
**Indoors**	**1,299**		**1,299**			
Mask Worn	1,274	98.1%	1,274	98.1%		
Mask Worn Correctly	1,191	91.7%	1,191	91.7%		
**Outdoors**	**1,509**		**701**		**808**	
Mask Worn	1,218	80.7%	640	91.3%	578	71.5%
Mask Worn Correctly	1,038	68.8%	566	80.7%	472	58.4%
**CSU**						
**Indoors**	**3,097**		**3,097**			
Mask Worn	3,042	98.2%	3,042	98.2%		
Mask Worn Correctly	2,894	93.4%	2,894	93.4%		
**Outdoors**	**128**				128*	
Mask Worn	96	75.0%			96	75.0%
Mask Worn Correctly	76	59.4%			76	59.4%
**National Aggregate**						
**Indoors**	**72,075**		**62,971**		**9,104**	
Mask Worn	69,504	96.4%	61,488	97.6%	8,016	88.0%
Mask Worn Correctly	64,471	89.4%	57,201	90.8%	7,270	79.9%
**Outdoors**	**28,278**		**19,557**		**8,721**	
Mask Worn	24,776	87.6%	17,576	89.9%	7,200	82.6%
Mask Worn Correctly	21,767	77.0%	15,492	79.2%	6,275	72.0%

*These observations were collected on the edge of the CSU campus; however, this environment was determined to be most like “Nearby, Off Campus”.

### Mask type, usage, and opinions on masking were similar at CU and CSU

**Fig. 2:**
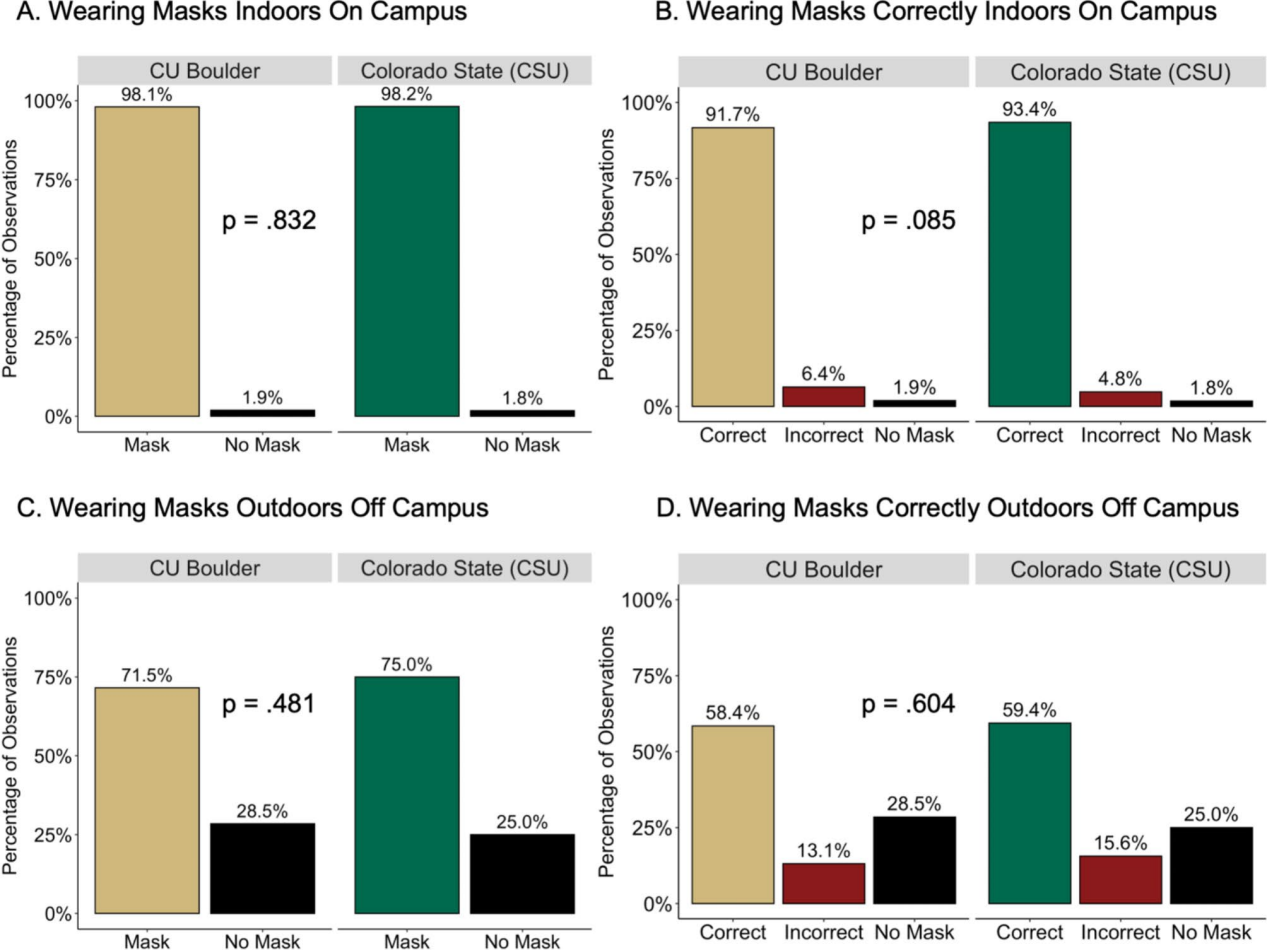
**Comparing Masking Indoors between CU and CSU and Comparing Masking Off Campus between CU and CSU. A–B** shows observations made indoors on campus at CU Boulder (CU; gold) and Colorado State (CSU; green). **(A)** Indicates the percentage of people wearing masks indoors on campus. **(B)** Indicates the percentage of people wearing masks correctly indoors on campus. **C–D** shows observations made outdoors off campus at CU and CSU. **(C)** Indicates the percentage of people wearing masks outdoors off campus. **(D)** Indicates the percentage of people wearing masks correctly outdoors off campus. Overall, there were no differences in masking behavior at CU compared to CSU as indicated by the p-value on each figure.

Cloth masks were the most popular at both Colorado universities, followed by surgical masks, while gaiters and N95-type masks were much less common. Across all CU observation sites, 88.7% of observed persons wore masks and 79.4% of observed persons wore a mask and wore it correctly (range across observation sites: 45.3–98.8%). Across all CSU observation sites, 97.3% of persons wore masks and 92.1% of people wore a mask and wore it correctly (range across observations sites: 59.4–100%). However, significant differences in masking behaviors by location were observed; proportions of masking and masking correctly varied whether observed indoors or outdoors and on campus or off campus (Table 2). As shown in Tables 1 and 2, this variability combined with the composition of observations by location and setting is a key factor in determining these overall rates of mask adherence by university and this variability makes it less informative to directly compare these aggregated percentages. For this reason, we compared mask behavior observed at specific locations and observation points with Chi-squared tests (Fig. 2) and confirmed the findings of no difference in mask usage (mask vs. no mask) between CU and CSU, using logistic regression.

**Fig. 3:**
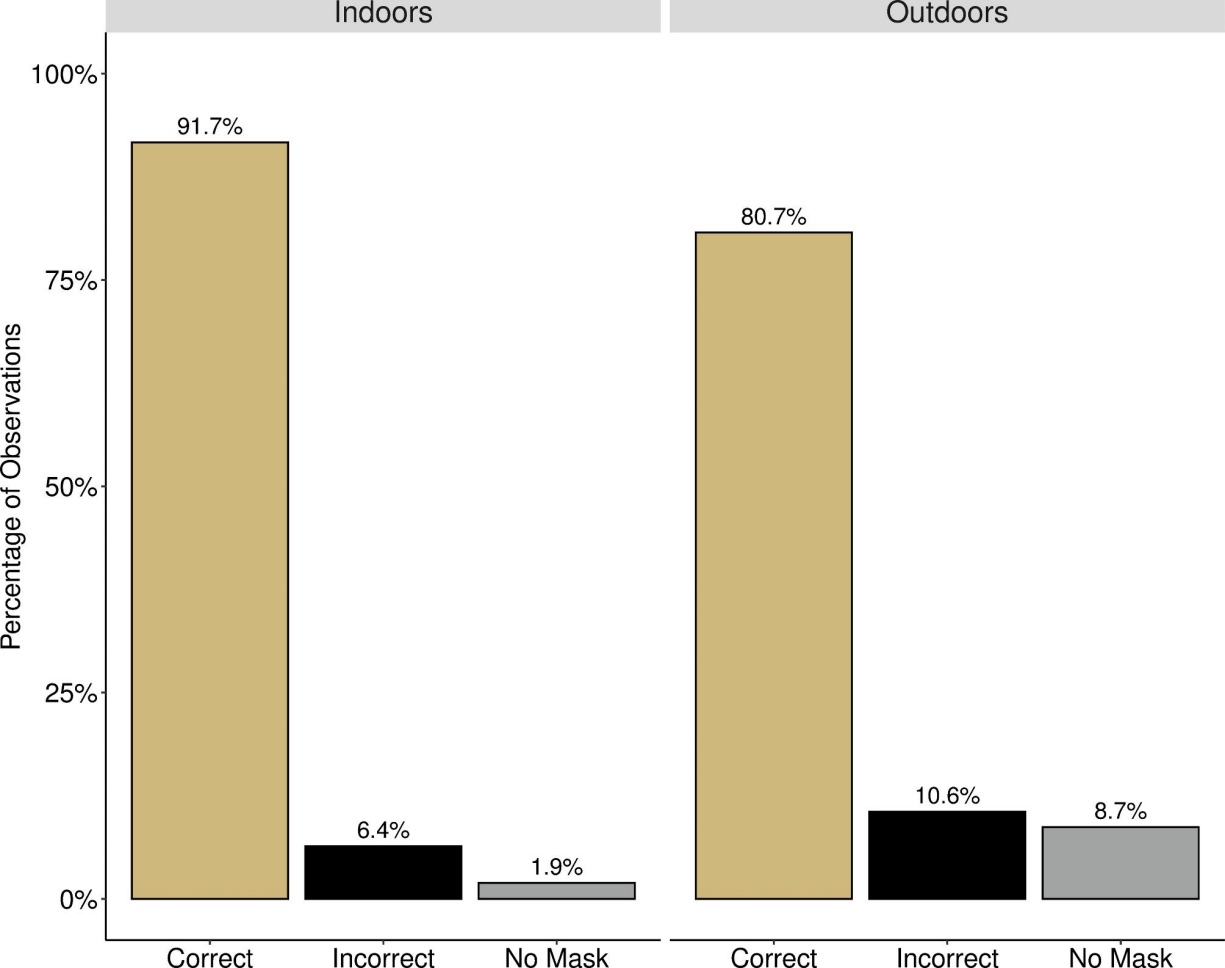
**Percentage of People Wearing Masks Correctly On Campus at CU.** Figure shows the observed differences between masking indoors (left) and outdoors (right) on campus at CU (p < .001). A Bonferroni correction was used to compare masking behaviors indoors vs. outdoors on campus. The frequency of correct masking was higher indoors compared to outdoors (p < .001). Conversely, the frequency of incorrect and no mask usage was higher outdoors compared to indoors (p = .006 and p < .001, respectively).

**Fig. 4:**
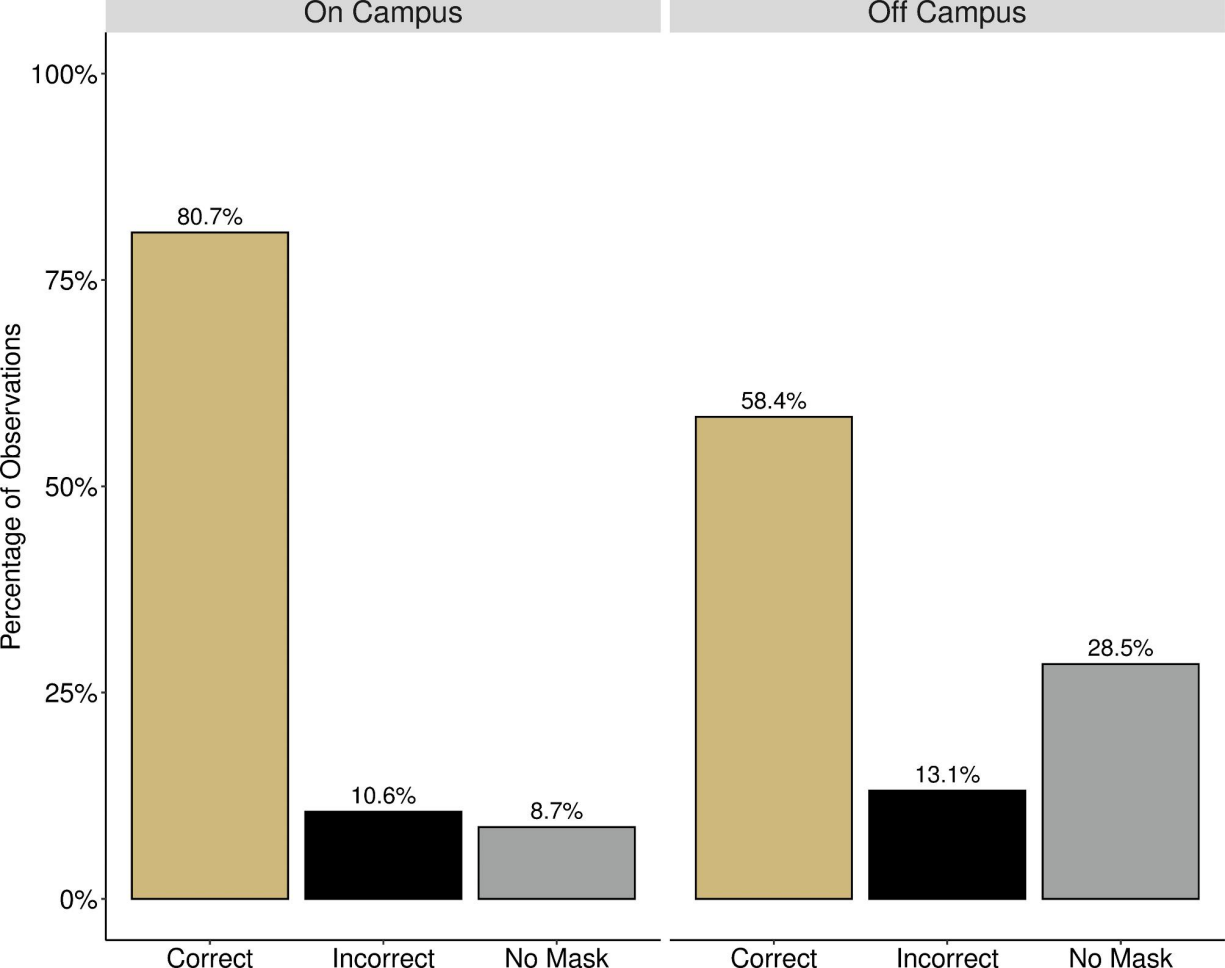
**Percentage of People Wearing Masks Correctly Outdoors at CU.** Figure shows the proportions of correct, incorrect, and no mask usage observed outdoors on campus (left) and outdoors off campus (right). At CU, there was a significant difference observed in masking behavior outdoors based on being on or off campus (p < .001). A Bonferroni post-hoc correction was used to compare masking behaviors on campus to off campus. The frequency of proper masking was higher on campus than off campus (80.7% vs. 58.4%, p < .001) and conversely the frequency of not wearing a mask was higher off campus than on campus (28.5% vs. 8.7%, p < .001). After correction there was no statistically significant difference between wearing a mask incorrectly on campus or off campus (10.6% vs. 13.1%, p = .754).

Correct mask use was significantly more common indoors than outdoors at both campuses. On campus at CU, masks were worn correctly 91.7% of the time indoors and 80.7% of the time outdoors (p < .001) (Fig. 3). At CSU, proper masking indoors was observed 93.4% of the time and outdoors 59.4% of the time (p < .001). However, all of CSU’s outdoor observations were also off campus. At CU, amongst outdoor observations there was a significant difference in the frequency of masks being worn depending on if the observed individual was on campus or off. Mask use was more prevalent outdoors at on-campus locations (91.3%) than at nearby off-campus locations (71.5%) (p < .001), as was correct mask use (80.7% vs. 58.4%, respectively; p < .001) (Fig. 4; Table 2).

At CU 46.3% of observations were performed indoors on campus where masks were worn 98.1% of time (Tables 1 and 2). By comparison, 96.0% of observations at CSU were indoors on campus where masks were worn at a nearly identical rate of 98.2% (Table 2; Fig. 2A). Additionally, the proportion of people observed wearing a mask and wearing it correctly indoors on campus were similar between CU (91.7%) and CSU (93.4%) (p = .085; Fig. 2B). Notably, this similarity between Colorado campuses also held for observations outdoors off campus. CU saw 71.5% of people wearing a mask and 58.4% of people wearing it correctly and CSU observed 75.0% (p = .481, Fig. 2C) of people wearing masks and 59.4% (p = .604, Fig. 2D) wearing them correctly. The similarities between masking behaviors at CU and CSU were confirmed using logistic regression while adjusting for observation location (i.e., on vs. off campus), observation point (indoors vs. outdoors) and observation week (weeks 1 through 8) and showed no differences in mask use between the institutions (p = .291) (**S2 Table**).

Overall, our observations on campus at both Colorado universities appear to be consistent with student opinions regarding masking that were gathered from survey responses. Regarding attitudes surrounding the importance of masking at CU, 92.9% of student respondents thought that wearing a mask can protect the health of others in the campus community. At CSU, 89.8% of student respondents indicated that they believe that wearing a mask at least partially reduces the spread of COVID-19 (10.2% responded that a mask does “not at all” reduce the spread of COVID-19). In response to questions about masking in different environments, CSU students reported that it was at least somewhat important to wear masks while in classrooms (83.6%) and moving around inside classroom buildings (81.4%), while fewer expressed that it was important to wear a mask while walking outside around campus (59.9%). When asked about the frequency with which students report mask use, 96.6% of CU respondents self-reported that they often wear a face mask when they are unable to maintain six feet of physical distance between themselves and others in public and 92.0% of CU respondents agree that they follow their campus’ policies related to COVID-19. However, only 15.3% of survey respondents at CU said they agree with the statement, “I believe students at my school are taking precautions to protect one another from COVID-19”. At CSU students had a more favorable perception of their peers’ behaviors. Specifically, 49.8% of respondents said that they think CSU students “often” or “very often” wear a mask in public even when it is not required and 4.4% said they think CSU students “always” wear a mask in public. Finally, 86.8% of students at CU reported that were it not for the pandemic they would prefer to take most of their classes in person.

### CU, CSU, and other participating institutes of higher education

Across all other institutes of higher education, cloth masks were still the mask of choice, but they were observed less frequently (61.5%) than at our Colorado campuses (63.3% & 69.0% for CU & CSU, respectively). Nationally, those observed may have opted for a gaiter over a cloth mask as gaiters were observed being worn more nationally (4.2%) than at both CU (2.6%) and CSU (2.3%). Collectively, 52 other institutes of higher education made more than 100,000 observations and 93.9% of observed persons wore masks, with 85.9% wearing them correctly. 62,971 of these observations (62.7%) were collected indoors on campus, while 8,721 observations (8.7%) were done outdoors off campus. This composition is relevant when comparing overall masking adherence because, as we have presented with CU and CSU (Table 2**)**, outdoor off-campus observations show the lowest masking rates of any environment. For comparison, 29% of CU’s observations and 4% of CSU’s observations were performed outdoors off campus.

At indoor locations on campus, masks were worn at other universities at a similar rate (97.6%) to Colorado institutes of higher education (p = .071), however CSU had a higher rate of masking correctly (93.4%) indoors than observed in aggregate across all other universities (90.8%) (p < .001). Additionally, CSU had a lower rate of masking incorrectly (4.8%) compared to the aggregate (6.8%) (p < .001). By comparison, CU was similar in the percent of those masking correctly indoors compared to the national average (91.7% vs. 90.8%, p = .488) and, as previously mentioned, there was no difference in correct mask use between CU and CSU indoors on campus. The percent of overall mask usage at outdoor locations on the CU campus was also similar to the on-campus aggregate average (91.3% vs. 89.9%, p = .242), as was correct mask usage at these locations (80.7% vs. 79.2%, respectively; p = .455). At outdoor off-campus locations 72.0% of the observed persons wore a mask and wore it properly across the aggregate of all other schools, which is more than the 58.4% at CU (p < .001) and the 59.4% at CSU (p = .007).

### COVID-19 case data and vaccination rates

**Fig. 5:**
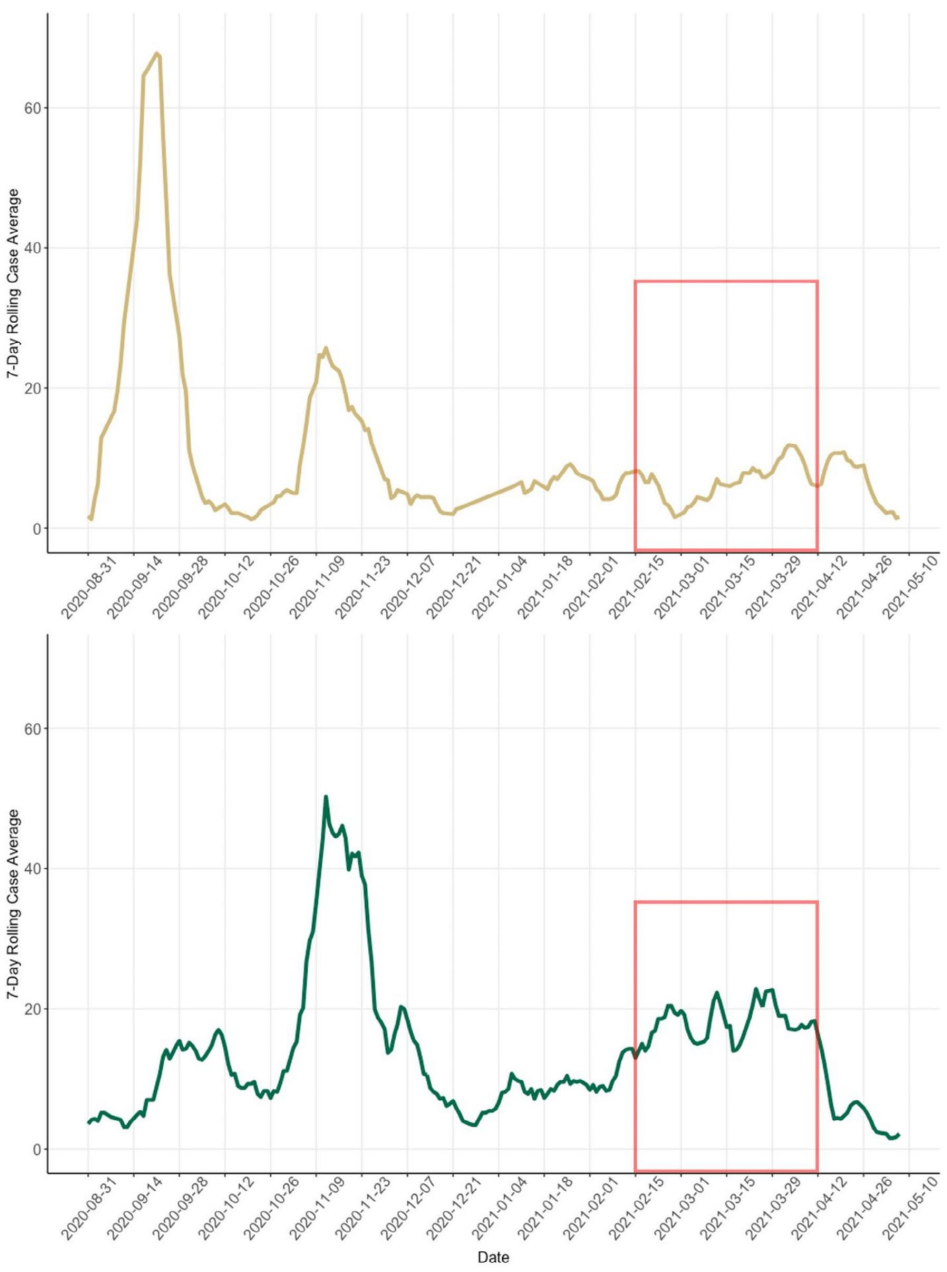
**CU Boulder (Gold) and CSU Fort Collins (Green) Confirmatory/Diagnostic PCR COVID-19 Cases (7-day rolling average).** Figure shows the 7-day rolling average of PCR diagnostic test confirmed COVID-19 cases over the academic year at CU (Gold) and CSU (Green). 7-day rolling average is calculated using sum of prior 7-day case counts divided by 7. The red outline is used to represent the period in which observations were performed at the universities

Both CU and CSU experienced their largest outbreaks of COVID-19 in the first semester of the 2020–2021 academic year (Fig. 5). Neither CU nor CSU returned to in-person instruction after the Thanksgiving holiday. CU saw relatively low case counts throughout the spring semester, in comparison to the spikes seen in the fall semester. During the observation period for the study, cases only surpassed a 10 case-per-day rolling average at the end of March / beginning of April. CSU also did not experience any surges in the spring semester near the magnitude of the fall case counts. During the mask observation period at CSU cases also remained relatively steady oscillating around the 17–20 new cases per day rolling average. At the time of the study there was not yet widespread vaccination amongst the campus population and counties were still focusing vaccination efforts on older adults and populations that are at higher risk. 12.1% of CU survey respondents (survey administered March 12–26, 2021) and 34.7% of CSU respondents (administered April 1–16, 2021) indicated they were already vaccinated. April 2 was the date that Colorado Department of Public Health and the governor extended vaccine eligibility in Colorado to the general public (ages 16 + years), otherwise college-age individuals would have had to be in a special employment or health-based risk group to be vaccinated prior to this date [49].

At CU, students and affiliates responding to the surveillance testing questionnaire who said they socialized multiple times per week outside their home without a mask were twice as likely to have tested positive for COVID-19 via saliva-based screening test (20% of these individuals had tested positive) as individuals who responded that they never socialize outside their home without a mask (10% of these individuals had tested positive).

## Discussion

In the current study, we investigated mask usage at the two largest university campuses in Colorado (CU and CSU) and compared rates with averages observed across 52 other participating institutes of higher education in the USA. At both Colorado schools, and across the other institutes, mask use rates were high during the Spring 2021 Semester and most people observed were wearing masks and wearing them correctly. CU’s on-campus indoor proportion of people masking correctly (91.7%) was in line with those observed at the other universities (90.8%), while CSU’s was slightly higher (93.4%). These rates of mask use were also in line with findings from the Fall 2020 MASCUP! Study (September to November 2020) which found that 92.1% of people observed wore masks correctly while indoors on campus across the six universities participating (one of which was CSU) [24]. This is a noteworthy consideration as it demonstrates mask use rates at participating institutes were high and consistent over a longer period spanning the 2020–2021 academic year. We also examined mask usage in the context of student opinions and perspectives using survey data collected on each campus and found that the proportion of people observed wearing masks correctly was similar to those who reported that wearing a mask could protect the health of others. Lastly, we found that both universities saw their largest spikes in COVID-19 cases in the fall semester of 2020, with no comparable spikes in cases during our observation period (Spring of 2021). Overall, these findings show high levels of mask adherence at Colorado’s two largest campuses and that masks were widely accepted. Further, our findings suggest that mask mandates encourage mask use compliance, which may help curb respiratory disease transmission at institutes of higher education and could help protect surrounding communities.

To date, a number of studies have observationally examined masking at colleges and universities in the United States and discussed the importance of this topic [24, 50–53]. The studies similarly found high rates of mask use on campuses. However, some studies did not perform observations off campus, failed to break down observations performed into indoor and outdoor locations (an important factor in mask use, as we discuss), or do not offer large representative samples of institutes of higher education nor present multi-institute aggregate data. Of studies that did have location specific data, they found that more than 95% of people observed on campus and indoors wore masks and masking was found to be significantly associated with being indoors, being on campus (vs. off campus), presenting as a woman, and the type of mask worn [24, 53]. Our findings are consistent with results from these studies where we found that face mask use was high (> 98%) and differed significantly between indoor and outdoor locations on campus, as well as outdoor locations off campus. Additionally, a study at an Indiana university found that people identifying as women correlated with higher belief in and self-reported adherence to preventative measures [54] and similar to surveys conducted at both Colorado universities, Indiana respondents reported higher adherence to preventative measures themselves than what they perceived for others. Masking behaviors by perceived gender were not reported in our study as demographic data of those observed was not collected during observation sessions.

Even though CU and CSU are in separate counties and 45 miles apart, the homogeneity of masking behavior between the two Colorado campuses was striking. In addition to the similar masking rates observed indoors on both campuses (CU: 91.7% CSU: 93.4%), 58–59% of people observed outdoors at nearby off-campus locations wore masks correctly. These rates are remarkably in line with the survey data: 92.9% of CU and 89.8% of CSU student respondents reported that masking can protect the health of others in the community and 60% of student respondents at CSU thought that it was important to wear a mask while walking outside. While surveys may be subject to social desirability bias [55], our objective and inconspicuous observations of mask use align with reported behaviors from surveys. This study is the first, to our knowledge, to report objective measurements of student mask use in the context of student opinions about masking and supports that student opinions may be representative of student’s actual behaviors regarding masking. However, our study was not designed to answer this question and further studies are needed to verify this relationship.

During the study, 53 of the colleges/universities, along with both Colorado campuses, required mask use on campus. Additionally, both Boulder and Larimer Counties had orders in place requiring masking at all indoor locations and limits on the number of people who can dine together at restaurants or congregate for gatherings [56, 57]. Over the Spring 2021 Semester, as the understanding of airborne transmission became more widespread, CDC updated guidance to emphasize the importance of ventilation and suggested that indoor mask use be prioritized. With this update, mask use outdoors was still recommended if six feet of physical distance could not be maintained [58, 59]. These guidelines may have impacted the higher rates of mask compliance observed indoors on campus vs. outdoors and shaped the student opinions on masking while walking outside. However, CU and other universities in the study still found higher rates of masking outdoors on campus compared to outdoors off campus. Additionally, the CDC found that in counties with mask mandates in addition to university mandates (n = 16 counties of 49 total counties participating) a higher percentage of people wore masks correctly overall (unpublished results from current pending CDC Spring ’21 MASCUP! submission). In Boulder (and Fort Collins) there were no city- or county-wide outdoor masking requirements (even when within 6 feet of others). Given this, observing lower rates of masking in outdoor off-campus areas in Boulder compared to outdoor on-campus areas could be reflective of a lack of policy requiring masking while off campus and within 6 feet of other people. Whereas higher rates of masking outdoors on-campus may have resulted from the university community interpreting mask requirements on campus to require that masks should be worn while outdoors as well as indoors. Supporting this, prior studies have also found that mask requirements or public health mandates increase mask usage compared to recommendations alone [23–26]. In combination, our findings and prior research support that individuals may be more likely to comply with prevention strategies when the campus and the surrounding local area have congruent policies.

At CU, CSU, and in the aggregate of all other participating schools, cloth masks were the most popular masks observed. Previous work by the CDC in the Fall of 2020 has established a relationship between people observed wearing masks correctly indoors and the type of mask they were wearing. Specifically, results from their study found that 96.8% of people wearing N95-type masks wore them correctly and 92.2% of people wearing cloth masks wore them correctly. By comparison, people wearing gaiters wore them correctly 86.8% of the time [24]. Given these findings, and the wide variability in protection based on mask type [60, 61], several universities have supplied N95/KN95 masks during case surges, including the Omicron variant period. Additionally, with the Omicron surge many national health departments (e.g., France, Germany, and Italy) moved to advising or requiring high filtration masks/respirators, such as N95s, for public settings and in schools while also recommending cloth masks no longer be used [62]. These findings also support that as N95 and KN95 masks are now more widely available, they may be the best choices for reducing disease transmission and should potentially be promoted on college campuses with future outbreaks or variants.

Masking has also been shown to be an effective tool in allowing schools to return to in-person instruction. A simulation study [63] and recommendation from the CDC [64] make a strong case that as a society we should consider prioritizing in-person schooling over (or at least alongside) many other activities — such as dining, bars, and casinos. Additionally, a study of K-12 schools found that physical distancing measures and symptom assessment in combination with masking and contact tracing led to very few reported in-school transmission events between students and no instances of child-to-adult transmission of SARS-CoV-2 [65]. While this study preceded the Delta and Omicron variants, it was also at a time where school age children did not yet have access to vaccination. Additionally, wearing masks on college and university campuses has been shown to be an effective prevention strategy in slowing the spread of COVID-19 [66, 67]. These study findings also agree with the data examined in the current study where CU’s Surveillance Testing Survey found an increased likelihood of testing positive for individuals who reported regularly socializing outside of their home without a mask.

With any observational study, the internal and external validity and generalizability are important considerations. We observed consistent rates of mask use over the 8-week observation period. We also observed similar rates of mask use between the Fall 2020 and Spring 2021 MASCUP! results. Lastly, we noted similarity between both Colorado Universities and the National Aggregate. Collectively, these observations suggest strong internal and external validity and suggest that our results are generalizable across universities. However, it is important to note that masking rates observed and attitudes about masking are less generalizable to settings outside of institutes of higher education and we do not intend to portray them as representative of the general public’s behaviors or beliefs. Thus, the aim of the current study is to report rates of mask use on college campuses from a specific time during the COVID-19 Pandemic and to share that these rates observed were high, especially on campus, and aligned with students’ opinions. These results serve as a benchmark and provide information about students’ opinions as a potentially modifiable factor to consider for public health messaging aimed at improving mask use/adherence on college campuses. If differences exist between larger universities and smaller institutes of higher education, then our results are biased towards representing larger institutions since both Colorado Universities are large schools (> 15,000 students). Lastly, it is important that as vaccination has become more widespread in the time since this study was conducted this may have affected student opinions about masking and has also changed our university policies about mask requirements, which would lead to differing rates of mask use now than observed during academic year 2020–2021.

As the ongoing effects and long-term implications of COVID-19 are still unknown and being uncovered, it is important to mitigate COVID-19 infection [68–70]. The CDC released findings that N95/KN95 masks provide the highest protection [61]; however they reiterated that the best mask is one that fits well and is worn correctly and consistently [71]. With the emergence of new variants of varying degrees of severity and transmissibility, understanding mask adherence on college campuses and policies that can create high compliance is essential. For example, the Delta and Omicron variants were each more infectious than the last, which resulted in spikes in infection, even with higher rates of immunity obtained through vaccination and prior infection. In the face of new variants or future diseases, masking remains a low-cost and effective prevention strategy to reduce disease transmission.

### Limitations

While we observed high rates of mask use on campus and lower levels of COVID-19 cases compared to the fall semester on Colorado campuses, this study was not designed to determine the impact of masking on disease transmission and thus the ecological observations about lower case counts and high rates of masking are presented as associations. Additionally, our observation methods aimed to capture the general population on and nearby campus, which would be primarily students; however, it is important to note that some campus observations likely included faculty and staff. Off-campus locations were more likely to include persons not affiliated with the university, though both off-campus locations were in areas with a high proportion of student traffic. Results from this study may not be representative of all colleges and universities in the United States. Though the term national average is used, this is not intended to imply a representative sample of all colleges nationally, but rather an average of the participating schools in MASCUP!. As mentioned, we recognize that this study is limited in generalizability to other populations outside of institutes of higher education and while masking in other settings is briefly discussed we do not represent our findings or these results to be reflective of any settings outside of the campus and nearby, off-campus environments. Lastly, while there is participation from some Historically Black Colleges and Universities in the study, as well as some smaller community colleges, these groups make up a small proportion of the overall universities represented in the national aggregate.

This observational study did not collect demographic data, which prevented us from examining any individual-level characteristics that may have impacted masking behavior and prevented us from determining if any individuals not wearing a mask may be exempt from mask use policies. In general, campus policy required that all people two years of age or older were required to wear a mask except for those who are hearing-impaired or otherwise disabled or who are communicating with someone who is hearing-impaired or otherwise disabled and where the ability to see the mouth is essential to communication. This and other medical exemptions to masking may exist and we cannot distinguish between various reasons for failing to adhere to masking guidelines.

Lastly, it is important to note that CU, CSU, and other schools participating in this study had reduced in-person instruction as a measure to curb disease transmission and reduce population density during the study period. The reduced number of people on campus (compared to full in-person operation) may have impacted students’ attitudes about masking and potentially impacted the observed masking rates at CSU and CU. The observations in this study were limited to the spring semester of 2021. Due to the aforementioned limitations, it is possible that these results may not be reproducible at a later date in the context of differing social and environmental influences and concomitant student opinions.

## Conclusion

As we progress through the “COVID-19 Era” and look to move forward as a society, the new knowledge, and discoveries of how to manage a highly contagious global pandemic will undoubtedly inform our future actions should another outbreak arise. The desire of students to have in-person education and the need for classes to be held on campus to prevent a loss in quality should be considered as universities decide how to proceed when faced with another pandemic or variant. The data presented here on masking behavior and student opinions about public health measures offer a benchmark for future reference in higher education. It is reassuring that students in Colorado were largely in favor of wearing masks in the Spring of 2021 and most viewed them as an important tool to limit the spread of COVID-19. Furthermore, actual indoor masking rates on both Colorado campuses were extremely high, indicating that personal behaviors aligned well with attitudes and that most students abide by policies that support in-person instruction. The current study indicates that masking was widely accepted, both in opinion and observed behavior, at institutes of higher education during the Spring of 2021, with corresponding high rates of compliance that may have aided in reducing disease transmission. Collectively, these findings support masking requirements and education about mask use benefits as key tools in supporting mask use on campus and likely reducing the spread of SARS-CoV-2 while enabling higher education to safely continue in person.

## Electronic supplementary material

Below is the link to the electronic supplementary material.

Supplementary Material 1

Supplementary Material 2

## Data Availability

The datasets used and/or analyzed during the current study are available from the corresponding author on reasonable request.

## List of abbreviations

ACHA: American College Health Association
aOR: Adjusted odds ratio
CDC: Centers for Disease Control and Prevention
COVID-19: Coronavirus Disease of 2019
CSU: Colorado State University Fort Collins
CU: University of Colorado Boulder
MASCUP!: Mask Adherence and Surveillance at Colleges and Universities Project
NCHA: National College Health Assessment
SARS-CoV-2: Severe acute respiratory syndrome coronavirus 2
