# Supplementary material for: High rates of observed face mask use at Colorado universities align with students’ opinions about masking and support the safety and viability of in-person higher education during the COVID-19 pandemic
Source: BMC Public Health. 2023 Feb 9;23:299. doi: 10.1186/s12889-023-15211-y (PMC9910780; doi:10.1186/s12889-023-15211-y)
Supplement: Supplementary file 1 — Supplementary Material 1 [file 12889_2023_15211_MOESM1_ESM.docx]

**Supplementary Material**

**
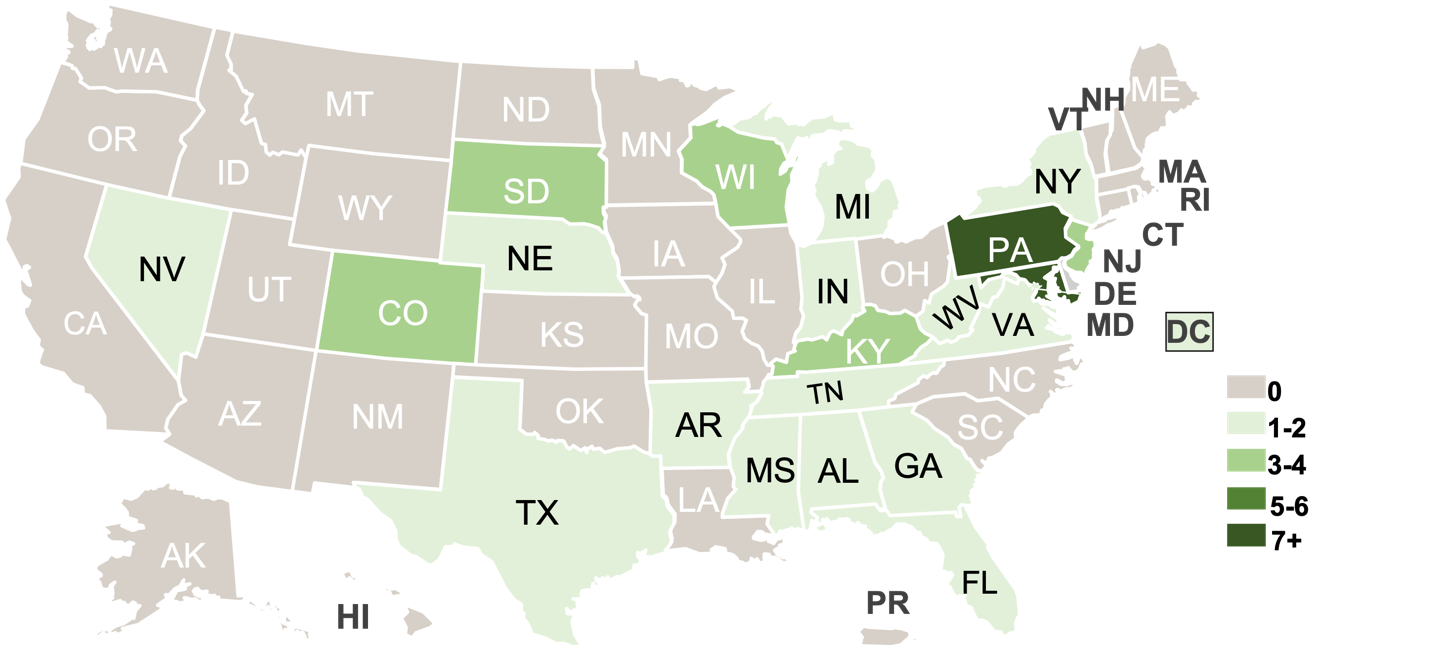
**

**S1 Fig. Participating Institutes of Higher Education by State.** Most participating states have 1-2 institutes, with 2 states having the largest number participating: Maryland (n=7) and Pennsylvania (n=9)

| *Variable* | *aOR* | *Lower 95% CI* | *Upper 95% CI* | *p-value* | *Coefficient Estimate* | *Std. Error* |
| --- | --- | --- | --- | --- | --- | --- |
| Ref: University of Colorado, Indoors, and On Campus | | | | | | |
| **Colorado State** | **1.323** | **0.775** | **2.200** | **0.291** | **0.280** | **0.266** |
| Outdoors | 0.253 | 0.165 | 0.386 | <.001 | -1.376 | 0.217 |
| Off Campus | 0.205 | 0.147 | 0.282 | <.001 | -1.583 | 0.166 |

**S2 Table. Odds of Wearing a Mask via Logistic Regression.** Adjusted odds ratios (aOR) and 95% confidence intervals of mask usage for university, inside vs. outside, and on campus vs. off campus using a logistic regression model. A higher aOR indicates an increased likelihood of wearing a mask.
